# Friendship networks and physical activity and sedentary behavior among youth: a systematized review

**DOI:** 10.1186/1479-5868-10-130

**Published:** 2013-12-01

**Authors:** Keri Jo Sawka, Gavin R McCormack, Alberto Nettel-Aguirre, Penelope Hawe, Patricia K Doyle-Baker

**Affiliations:** 1 Department of Community Health Sciences, Faculty of Medicine, University of Calgary, Calgary, Alberta, Canada; 2 Department of Pediatrics, Faculty of Medicine, University of Calgary, Calgary, Alberta, Canada; 3 Faculty of Kinesiology, University of Calgary, Calgary, Alberta, Canada

**Keywords:** Friendship, Social network, Physical activity, Sedentary, Obesity

## Abstract

**Background:**

Low levels of physical activity and increased participation in sedentary leisure-time activities are two important obesity-risk behaviors that impact the health of today’s youth. Friend’s health behaviors have been shown to influence individual health behaviors; however, current evidence on the specific role of friendship networks in relation to levels of physical activity and sedentary behavior is limited. The purpose of this review was to summarize evidence on friendship networks and both physical activity and sedentary behavior among children and adolescents.

**Method:**

After a search of seven scientific databases and reference scans, a total of thirteen articles were eligible for inclusion. All assessed the association between friendship networks and physical activity, while three also assessed sedentary behavior.

**Results:**

Overall, higher levels of physical activity among friends are associated with higher levels of physical activity of the individual. Longitudinal studies reveal that an individual’s level of physical activity changes to reflect his/her friends’ higher level of physical activity. Boys tend to be influenced by their friendship network to a greater extent than girls. There is mixed evidence surrounding a friend’s sedentary behavior and individual sedentary behavior.

**Conclusion:**

Friends’ physical activity level appears to have a significant influence on individual’s physical activity level. Evidence surrounding sedentary behavior is limited and mixed. Results from this review could inform effective public health interventions that harness the influence of friends to increase physical activity levels among children and adolescents.

## Background

Physical activity plays a vital role in the health of children and adolescents [1]. Along with a high caloric diet, low levels of physical activity and increased participation in sedentary leisure-time activity are two important lifestyle behaviors that have contributed to the increased prevalence of overweight and obesity among youth and adults [2,3]. In children and adolescents, overweight and obesity are associated with an increased risk of high blood pressure, dyslipidemia, impaired glucose tolerance, cardiovascular disease, and type II diabetes [4,5]. Furthermore, overweight children are highly likely to become overweight adults, which may reflect the tracking of obesity-risk behaviors (i.e., physical activity and diet) from childhood into adulthood [4,6].

The social environment comprises the physical surroundings, social relationships and cultural milieu within which people function and interact [7]. It has been shown to influence obesity-risk behaviors in adults [8,9]; those reporting low social support from family and friends are more likely to be insufficiently active for health benefits compared to those with high levels of social support [8]. The social environment also plays an important role in relation to children’s physical activity and sedentary behavior. The social environment of children includes the influence of parents, siblings, friends, neighbors, teachers, and coaches [10,11]. While parents are the most important source of influence in early-life, parental influence on their child’s day-to-day behavior becomes less evident as the child matures [12,13]. Children and adolescents spend a significant portion of their time at school with friends and peers. Evidence suggests that the dietary behavior of a friend or group of friends influences the dietary behavior of the individual [14], with similar results observed for sports participation [14] and sedentary behavior [15].

The pathways by which behaviors may be similar among groups of friends during childhood, however, are complex. Similar behaviors among friends likely reflect the processes of homophily or selection (i.e., an individual with certain behaviors seeking out others who also share similar behaviors) and peer influence or peer contagion (i.e., the influence of friends’ behaviors causing changes in an individual’s behavior) [16]. Several mechanisms may explain the processes of peer influence and contagion on physical activity and sedentary behavior including: behavioral modeling (i.e., observing a peer perform a behavior leading to increased motivation to perform a behavior); peer pressure (i.e., direct attempts to impose a certain behavior on a peer); group norms (i.e., the underlying attitudes and behaviors shared among a group of peers), and; co-participation (i.e., undertaking a behavior with a peer potentially contributing to behavioral reinforcement) [17,18].

Social network analysis or sociometry [19] provides a means of studying the inter-relationships among friends themselves and does not rely on an individual recalling or reporting the behavior of his/her friends or peers. Social network analysis is a quantitative method for assessing the structure and patterns of the ties or relationships among a set of entities (e.g., people or organizations) [20]. It can provide information about an individual’s local relations (e.g., who he or she is friends with) and network position (e.g., whether he or she is centralized within a given network) as well as measures of the entire network itself (e.g., number of connections between people, and degrees of separation [16]). In child and adolescent health, social network analysis has been used extensively to investigate behaviors such as smoking, substance use, and delinquency in relation to individual-level network measures [21-24]. For example, popularity, or being nominated as a friend by many others, is associated with higher odds of drinking alcohol among thirteen and fifteen year olds [21], while substance use is associated with receiving fewer friendship nominations [25]. Smoking [26], delinquency [24], substance abuse [21], and depression [27] studies that have used social network analyses suggest that the attitudes and behaviors of adolescents influence the attitudes and behaviors of others in their friendship networks (i.e., peer contagion). Moreover, the influence of peer contagion might also be gender-specific. Mercken et al. [28] found that teenage girls, but not boys, were influenced by their peer group to initiate smoking, while delinquent behavior in friends may be more influential in boys than girls [29].

Regarding physical activity, some evidence derived from social network analysis suggests that higher physical activity levels within friendship groups could be associated with higher levels of participation among individual group members [30]. Much of this evidence is based on individual-level or ego-network measures (i.e., a direct link between individuals) rather than an individual’s position in the network of a class or school or the characteristics of the networks themselves. Furthermore, similar to other behaviors, there is preliminary support for gender-specific relationships between individual measures of friendship networks and physical activity. Jago et al. [31] found that moderate-to-vigorous physical activity of boys’ best friends, but not girls’ best friends, was positively associated with an individual’s moderate-to-vigorous physical activity.

Little is known about how specific network ties (i.e., local relations) and specific network roles (i.e., positions within the network) might influence physical activity and sedentary behaviors among children and adolescents. For example, a non-reciprocated friendship nomination (i.e., person ‘A’ says ‘B’ is my friend, but person ‘B’ does not say ‘A’ is my friend) may have a different influence on behavior compared to a reciprocated nomination. The concept of reciprocation in a friendship network can indicate the presence of strong ties (reciprocated nomination) and weak ties (non-reciprocated nomination) between individuals. Strength of ties may also be related to degree of friendship separation (i.e., friend of a friend) [32], or intimacy of friendship (i.e., first nominated friend, second nominated friend) [33]. Specific roles within a network may also influence behavior, such as being an isolate (i.e., no ties to other individuals) or liaison (i.e., providing ties between groups within a network) [26]. While studies have identified relationships between specific network roles (e.g., isolates) and smoking [21], as well as network characteristics (e.g., density) and delinquency [24], these relationships in the physical activity and sedentary behavior literature are still poorly understood. Knowledge of the dynamics of friendship networks in relation to physical activity and sedentary behavior could be useful for informing health promotion interventions within social settings (i.e., schools).

A recent systematic review found strong similarities between a child or adolescent’s level of physical activity and that of his/her close friends and wider peer group, but limited evidence on the role of social networks in influencing sedentary behavior [30]. These authors, along with others [34], suggest that better interventions may come from better understanding of friendship networks and behavior. To do so, however, requires a deeper understanding of the psychology and sociology of networks, such as who should be recruited to interventions and how experiences and messages can be amplified (or diluted) across the group [35]. School-based, peer-group interventions in drug use lacked this sophistication, with consequent modest or negligible effects [36].

The purpose of this review was to expand and reassess the conclusions of a previous synthesis [30] by undertaking a systematized literature review of studies examining the association between friendship networks and both physical activity and sedentary behavior. A systematized review encompasses several, but not all aspects of a full systematic review [37]. The objectives of this review were to: 1) examine the association between a friend’s level of physical activity and sedentary behavior and an individual’s levels of physical activity and sedentary behavior; 2) determine if the number of friends a child or adolescent has influences his/her own physical activity or sedentary behavior, and; 3) identify and differentiate the effects of different types of social network measures, for example, network ties and positions, that are potentially associated with physical activity and sedentary behavior, especially as they operate at gender-specific levels.

## Method

### Database search and study inclusion

To identify studies for possible inclusion in our review, seven scientific online databases covering the medical, (MEDLINE, PubMed, CINAHL), kinesiology (SPORTDiscus), education (ERIC), sociology (SocINDEX), and psychology (PsycINFO) fields were searched. Search terms and phrases were combined and reflected the population of interest (i.e. child, preteen, adolescent, student, teen, boy, or girl), the exposure (i.e. social network, friend, peer, or social group), and the outcomes (i.e., physical activity, play, sport, exercise, sedentary, inactivity, or leisure). Searches within each database were restricted to English language, peer-reviewed, and primary studies. No restrictions were placed on year of publication. Databases were searched in June, 2012. Our broad search strategy resulted in 21,354 articles. KJS initially reviewed these titles and removed duplicates, non-journal articles and irrelevant titles. The remaining abstracts (n = 1,676) were reviewed in detail by KJS and a random sub-sample (n = 300) were reviewed by GRM to ensure scientific rigor (88.3% overall agreement).

Seventy-one articles were identified to undergo a full paper review and were read in detail by KJS and GRM. Studies eligible for this review must have included: children or adolescents aged six to eighteen years of age; a measure of a participant’s friendship network through either friendship nominations (i.e., participant nominating friends from a class list) or friendship rating (i.e., participant indicating whom they prefer to play with most), and; a measure of physical activity or sedentary leisure-time activity (i.e., direct observation, motion monitors, direct or indirect calorimetry, doubly-labeled water, parent proxy, or self-report) for both the participant and the participant’s nominated friends. Studies that utilized a general social support measure (i.e., how often does your best friend encourage you to exercise?) were excluded. We also excluded studies that used participant’s proxy measure of friend’s physical activity or sedentary behavior. This was to ensure that each participant identified his or her friends (whom also participated in the study), and that each participant recorded his or her own level of physical activity and sedentary behavior. Final inclusion of each study was based on consensus of two authors (KJS and GRM). To broaden our search, reference lists from included studies were scanned to further identify potential studies.

### Data extraction and analysis

From each included study, information regarding study design, sample size, participant characteristics, description of friendship network or friendship rating measure, physical activity and/or sedentary behavior, confounders, and study findings were extracted and tabled. The most robust results from each study were included (e.g., findings based on adjusted estimates would be presented instead of findings based on unadjusted estimates if both were presented within a single study). Factors affecting study validity including sample design, sample size, response rate, control for confounders, and method of physical activity or sedentary behavior measurement were appraised and synthesized, along with study results of the relationships between friendship networks and physical activity and sedentary behavior. Information regarding the use of a theoretical framework or model, where reported, was also extracted from each article.

## Results

**Figure 1:**
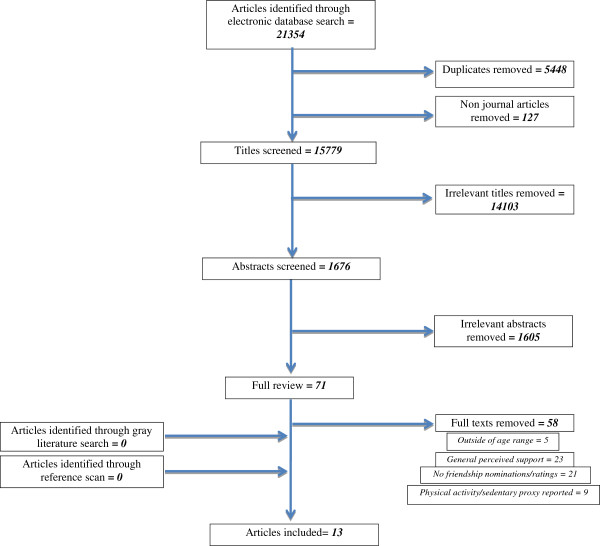
Flow diagram of article search and selection.

A total of thirteen studies were included in this review, four [38-41] of which were not included in the previous review [30] (Figure 1).

### Characteristics of studies reviewed

The reviewed studies included children and adolescents ranging from six to eighteen years of age (Additional file 1: Table S1). One study [42] included girls only, while the other studies had approximately equal proportions of boys and girls. Eleven studies reported response rates ranging from 58.6% to 93% [14,15,31,38,39,41-46]. Of those, six had response rates of 80% or lower [31,39,44-47]. The geographical location of studies included Australia (n = 4), the United States (n = 3), the United Kingdom (n = 2), Canada (n = 1), Estonia (n = 1), Finland (n = 1) and Norway (n = 1). All of the studies occurred within a school or after-school setting.

Nine studies were cross-sectional [14,15,31,38,41,42,44,46],[47], while the remaining four were longitudinal [39,40,43,45]. Length of follow-up time for the longitudinal studies ranged from one to five years. Seven studies measured physical activity using self-administered questionnaire [15,38,39,41,43,46,47], four via accelerometer [31,40,44,45], one via pedometer [42], and one via face-to-face interview [14]. Three studies [14,15,46] also assessed the amount of sedentary leisure-time activities, which included hours per day of watching television and videos, playing video or computer games, or using the Internet. For the participant’s friendship network measure, all but two studies [45,47] used participant nominated friends and best friends in their class, grade, school, or after school program. Livesey et al. [47] asked children to rate how much they liked to interact during play with other children included in the sample, while Ommundsen et al. [45] used children’s preferences to play and work with other children in the study to create a socio-metric status score for each participant. Further, Strauss and Pollack [46] measured participant’s five best male and five best female friends, and determined the relationship between this measure of popularity and both sports participation per week and hours of television or video watching per day.

Twelve studies statistically controlled for at least one confounding variable, while Schofield et al. [42] did not report controlling for confounders. Across these twelve studies, demographic variables were controlled for, including age and gender. Six studies adjusted for weight status [14,15,31,40,45,46]. Several studies also adjusted for socioeconomic factors including parent socio-economic status, parent education level, and/or participant pocket money [14,15,39,41,44-46]. Only three studies [38,41,43] explicitly stated the use or application of a theoretical framework or model with regard to their study design or interpretation of findings. De la Haye et al. [43] used the Theory of Planned Behavior, with particular focus on perceptions of peer (subjective) norms as a key mechanism of peer influence. These authors however, noted that Self-Perception Theory, where an individual becomes aware of their own psychological and emotional states based on the individual’s observation of their own behaviors, might have provided a better explanation of their results. Raudsepp and Viira [41] used Social Learning Theory, with particular focus on the concept of behavioral modeling to explain their significant findings whereby best friend’s physical activity was positively associated with an individual’s physical activity. Yli–Piipari et al. [38] applied the expectancy-value model, which emphasizes personal values and expectancies, as a means to help define socialization and friendship interactions and further explain similarities in physical activity behavior among groups of friends.

In terms of friendship nominations, one study used only reciprocated nominations [39], while others used both reciprocated and non-reciprocated nominations [15,31,38,40,42-44]. Two studies [14,41] did not indicate whether they used reciprocated and or non-reciprocated nominations. For studies that specifically examined popularity (e.g., the number of times a participant was nominated as a friend) or a socio-metric measure (e.g., preference to play with particular individual), reciprocation of a friendship nomination was not needed as this measure is based on how many times a participant was nominated [45-47].

### Associations between friendship networks and physical activity

Of the ten studies [14,15,31,38-44] that measured close friends’ or friendship groups’ physical activity levels, all found some evidence that levels of physical activity among friends was associated with the level of physical activity of the individual (Table 1)*.*

**Table 1:** Summary of the associations between friendship networks and physical activity and sedentary behavior across reviewed studies

		***Associations with physical activity***			***Associations with sedentary behavior***		
		**Positive**	**Null**	**Negative**	**Positive**	**Null**	**Negative**
**Boys**	Close friends	[15], [39]^a, b^, [31], [41]	[15], [31]		[15]	[15]	
	Friendship group	[38]*					
	Popularity	[15]	[15], [47]	[45]^b^		[15]	
	Friendship selection						
**Girls**	Close friends	[15], [39]^a, b^, [41], [42]*^a^	[15], [39]^a^ , [31], [41], [42]*		[15]	[15]	
	Friendship group	[42], [38]					
	Popularity	[45]^b^	[15], [47]		[15]	[15]	
	Friendship selection						
**Boys and girls**	Close friends	[43]^b^, [40]^b^					
	Friendship group	[14], [44]	[44]			[14]	
	Popularity	[45]^b^, [46]	[40]	[45]			[46]
	Friendship selection	[43]^b^	[40]^b^				

*Note.* *Associations significant at p <.10. All other associations significant at p <.05.

^a^ = reciprocated nominations only.

^b^ = longitudinal analysis.

*Close friends*: Physical activity or sedentary behavior of nominated best friend or close friends. *Friendship group*: Average physical activity or sedentary behavior of nominated friends. *Popularity*: Higher number of received friendship nominations or a higher measure of friendship rating/status (i.e., number of nominations received for preference to play with). *Friendship selection*: Individual choosing a friend based on similarities with his or her own physical activity or sedentary behavior.

#### Popularity, socio-metric status, and physical activity

Five studies [15,40,45-47] assessed popularity level or socio-metric status, and physical activity level of the individual and found differing results. Strauss and Pollack [46] found that a higher count of friendship nominations was associated with higher sports participation. This supported De la Haye et al.’s [15] finding that boys who played more organized physical activity tended also to be the most popular among school friends. In contrast, Gesell et al. [40] and Livesey et al. [47] did not find any significant association between popularity level and physical activity among boys and girls. Ommundsen et al. [45] found that higher total accelerometer counts were correlated with lower socio-metric status in grade one children. Furthermore, in a longitudinal analysis, Ommundsen et al. [45] found that, for girls, higher total accelerometer counts in grade one were associated with a higher socio-metric status in grade four, while for boys, higher total accelerometer counts in grade one were associated with a lower socio-metric status in grade four.

Three longitudinal studies [39,40,43] assessed the change in participant’s physical activity level over time, and all found that participants’ level of physical activity significantly changed over time to emulate friends’ higher levels of physical activity. Two longitudinal studies [40,43] also examined whether participant’s friendship selection was based on physical activity levels; De la Haye et al. [43] found that friendship selection was significantly influenced by similarities in physical activity levels, whereas Gesell et al. [40] did not.

#### Network position and physical activity

Schofield et al. [42], although not adjusting for other factors, found that a higher pedometer step count for girls’ first nominated *reciprocated* friends was moderately correlated with a high pedometer step count for the individual; however, first *non-reciprocated* friend’s step count was not correlated with an individual’s step count. Moreover, this study also found that the correlation between step count and nominated friends attenuated as friend’s intimacy (i.e., second and third nominated friend) decreased regardless of whether or not the nomination was reciprocated [42]. Macdonald-Wallis et al. [44] measured degree of friendship separation, and found that the correlation of moderate-to-vigorous physical activity and counts per minute among friends was strongest with more immediate friendships (i.e., no separation via another person). Beyond nomination reciprocation and degrees of separation, studies did not include measures of local network roles (e.g., isolate, liaison), nor did they examine network-level measures (e.g., density, centrality).

#### Gender differences between friendship networks and physical activity

Six studies [15,31,38,39,41,45] reviewed found differences between the influence of friends on physical activity and sedentary behaviors of boys and girls. Boys tended to be more active, and were more likely to be influenced by the physical activity behaviors of their friends compared to girls. For example, Jago et al. [31] and Raudsepp and Viira [41] found that boys’ friend’s moderate-to-vigorous physical activity was associated with individual’s moderate-to-vigorous physical activity, but this association was not statistically significant for girls. Denault and Poulin [39] found that, for boys, a higher level of friend’s sports participation was associated with a higher level of individual sports participation.

### Associations between friendship networks and sedentary behavior

Three studies [14,15,46] examined the association between friendship networks and sedentary behavior and found contradicting results (Table 1). Ali et al. [14] found no association between the weekly hours of television and video viewing of nominated close friends’ and an individual’s television and video viewing. In contrast, De la Haye et al [15] found significant positive associations between friends’ video/computer gaming and Internet use and individual’s (girls only) video/computer gaming and internet use in three separate age-based networks (school 1/grade 8; school 2/grade 8; school 2/grade 9). A positive association was also found for boys for the school 2/grade 8 network [15].

#### Popularity, socio-metric status, and sedentary behavior

Strauss and Pollack [46] found that as an adolescent’s (boys and girls combined) popularity increased, they spent less time per day watching television.

#### Network position and sedentary behavior

There were no studies that examined differences in reciprocated or non-reciprocated friendships, degree of separation, specific network positions or network characteristics.

#### Gender differences between friendship networks and sedentary behavior

One study stratified their results by gender [15]. De la Haye et al. [15] found an association between higher levels of girls’ friends’ video/computer gaming and Internet use and higher levels of individual video/computer gaming and Internet use in all three networks examined. Boys associations were only present in one network [15]. Contrary to Strauss and Pollack [46], De la Haye et al. [15] also identified a small but significant association between a girl’s popularity (i.e., greater count of friendship nominations) and increased level of participation in video/computer gaming and Internet use.

## Discussion

Friendship networks are associated with physical activity among children and adolescents, with some, albeit less, evidence suggesting that friendship networks might also be associated with sedentary behavior. Our findings confirm evidence from a previous review [30] which showed that peer networks have a greater influence on physical activity and sedentary behavior for boys compared with girls. This observation is strengthened by more longitudinal evidence, lending weight to the peer contagion models of physical activity (i.e., after becoming friends, behavior become similar) as opposed to the peer selection model (i.e., adolescents choosing friends who have similar behavior to themselves at the outset). This review identified a lack of explicit use of theoretical frameworks in studies to date.

The differential influence of friendship on physical activity for boys and girls may reflect differences in attitudes towards physical activity and differences in peer social norms [48]. Moreover, boys generally have higher levels of fitness and physical activity participation compared with girls [49,50]. Higher levels of physical activity in and of itself might provide more opportunities for co-participation and modeling (i.e., an individual witnessing another individual being active and may be therefore motivated to participate in the same activity). Another, albeit weaker, explanation could be that the faster rate of maturity among girls, on average, might result in girls developing a more concrete set of values sooner and therefore less likely to conform to group norms [51]. Gender differences have also been identified for diet, with boys’ friends being more alike in their consumption of high caloric foods than girls’ friends [15]. This could suggest that gender-specific approaches to promoting healthy weight might be needed, especially if the primary vehicle for the intervention is the friendship network. However, more research is needed to identify which social mechanisms might be more influential in determining physical activity and sedentary behavior for boys and girls.

Similarities in friendship network behaviors can be both the result of social influence, where children or adolescents adopt behaviors based on the attitudes and behaviors of friends within a network, or a result of friendship selection, whereby individuals select friends that share similar interests, attitudes, and behaviors [15]. The processes of peer influence and peer selection are found to be associated in other health behaviors in the adolescent population including smoking [52] and delinquency [53]. Disentangling these pathways is difficult based on cross-sectional study design, which includes the majority of studies reviewed here. While cross-sectional studies are able to tell us whether a relationship exists between a friendship network and an individual’s behavior, the direction of causality cannot be ascertained. The longitudinal studies in this review offer key information in terms of the influence of friendship networks on physical activity as they allow potential causal pathways to be extricated. Three of these studies [39,40,43] found that an individual’s physical activity level changed over time to become more similar to a friend’s higher level of physical activity, while the fourth longitudinal study [45] found a positive relationship for girls’ socio-metric status in grade four and accelerometer counts in grade one. These results provide evidence to support a causal pathway, where friends influence an individual’s physical activity level (i.e., peer contagion). This friendship influence could be a result of social norms. Pressure from peers to conform to group norms is a strong motivator for behavior adoption or maintenance, and is often combined with negative consequences, such as social isolation, if behaviors are not adopted [18]. Future research that assesses reasons for choosing friends will assist in understanding the factors (i.e., friendship selection versus friendship influence) that influence similarities in health behaviors across friendship networks.

Studies included in this review used mainly ego-based networks, where participants were asked to self identify and nominate their best or close friends; this compared to using complete friendship networks, where participants are given a full class or school list and asked to nominate their friends, thereby allowing the identification of each participant’s role within a friendship network. Previous research has recognized the importance of friendship network roles and characteristics (e.g., density, centrality) in relation to health behaviors in youth [21,24,26,54]. A review by Seo and Huang [54] found that isolates (i.e., no ties to other individuals [19]) were more likely to be smokers compared to clique members (i.e., members of a group of at least three individuals, where all three individuals are linked through friendship nominations [19]), and further identified that non-smoking adolescents were more likely to become smokers if they belong to a smoking clique. There were no studies in our review that investigated the specific roles within a complete friendship network, such as liaisons (i.e., providing ties between groups within a network [19]) or isolates. Examining the relationship between isolates and physical activity and sedentary behavior may have important health implications, as one study [46] found that decreased friendship nominations was associated with greater television and video viewing. Furthermore, liaisons are characterized as having a strong degree of interaction among several cliques, and therefore may be a useful mechanism to promote physical activity to a greater number of individuals.

Studies included in this review did not measure the length of friendship, frequency of friend contact, or context in which friends normally interacted (e.g., playing at recess or after school). The former measures can indicate the strength of bond between two individuals, while the latter measure may have a specific impact on a friend’s influence on sedentary behavior, as sedentary leisure-time activities generally occur outside of the school setting. As well, the level of influence friends have on one another’s behavior might depend on whether the context and activities are organized or non-organized (e.g., sports vs. unstructured play). Stronger bonds, as seen through reciprocated friendship nominations, have a greater impact on physical activity levels as compared to non-reciprocated friends [42]. Accounting for the quality or strength of friendship bonds in addition to friendship ties may provide greater insight into the mechanisms explaining peer influences on physical activity and sedentary behavior.

As with any review, the issue of publication bias should be considered when interpreting our findings. This review did not objectively-assess the scientific quality of each included study nor weigh findings based on their validity (i.e., using a validity assessment). Noteworthy, was that only three studies explicitly mentioned the use of a specific theoretical framework or model. Integration of the mechanism of peer selection or contagion within existing social cognitive models of behavior may provide greater understanding regarding peer influence on physical activity and sedentary behavior. At a minimum, future studies should describe the theoretical frameworks informing their methodologies and interpretation of results.

Despite undertaking a broader search of literature to identify studies, we found only four additional studies not included in a review completed approximately two years ago [30]. Nevertheless, these additional studies contributed to current knowledge – for example, one study provided additional support for gender differences with regard to peer influence as well as the association between peer influence and physical activity intensity [41], and two studies provided longitudinal evidence showing emulation of friends physical activity behavior over time [39,40]. However, our review identified several gaps in current knowledge, not previously identified, including the lack of evidence regarding the association between specific social network ties, roles, positions, and characteristics and physical activity and sedentary behavior, the dearth of studies incorporating measures strength or quality of peer relationships, the lack of details regarding theoretical frameworks and models, and the need for more longitudinal study designs. Given that there are only thirteen published studies on this topic suggests that our understanding of the role of social networks on physical activity and sedentary behavior among youth is in its early stages and that this topic demands more research attention.

Findings from this review provide support for a relationship between friend’s physical activity and an individual’s physical activity in children and adolescents, but findings for sedentary behavior are mixed. Harnessing the influence of friendship to increase physical activity levels and decrease sedentary leisure-time activity would have a beneficial impact on reducing the current prevalence of overweight and obese youth through an increase in energy expenditure. More research examining sedentary behavior among children is needed, including investigation of virtual peer networks that result from on-line gaming, as well as the influence of networks outside of the school setting (e.g., family, sports teams, camps, social clubs) on obesity-risk behaviors.

## Competing interests

The authors declare that they have no competing interests.

## Authors’ contribution

KJS/GRM/ANA conceived the study. KJS lead the database search, article selection, synthesis, and drafting of the manuscript. GRM/ANA assisted in article selection and synthesis. All authors contributed to the interpretation of findings and writing of the manuscript. All authors read and approved the final manuscript.

## Supplementary Material

Additional file 1: Table S1 Characteristics of reviewed studies. Click here for file
