# Supplementary material for: Friendship networks and physical activity and sedentary behavior among youth: a systematized review
Source: Int J Behav Nutr Phys Act. 2013 Dec 1;10:130. doi: 10.1186/1479-5868-10-130 (PMC4220781; doi:10.1186/1479-5868-10-130)
Supplement: Additional file 1: Table S1 — Characteristics of reviewed studies. [file 1479-5868-10-130-S1.docx]

| Supplement table. Characteristics of reviewed studies |
| --- |

| **Author(s)** | **Study Design (Country) and theoretical frameworks/models** | **Sample Size (Response Rate)** | **Sample Characteristics** | **Independent Variable** | **Dependent Variable** | **Confounding Variables** | **Findings** |
| --- | --- | --- | --- | --- | --- | --- | --- |
| Ali et al. [14] | Cross-sectional (United States)  Theoretical framework/model: not stated | n= 3898 (88.6%) | 16.26 ± 1.56 yrs of age; 51.30% girls | Researcher conducted in-home interview; a) Exercise, sports playing, TV watching of nominated close friends within school | Researcher conducted at home interview; a) Exercise (< 3 or ≥ 3 times/wk), b) Playing an active sport (Y/N), c) Television/video viewing (hrs/wk) | Demographics, weight status, socio-demographics, parental variables | Friend's exercise associated with individual’s exercise (+); Friend's sports participation associated with individual's sports participation (+); Friend's TV/video viewing not associated with individual's TV/video viewing (n.s). |
|  |  |  |  |  |  |  |  |
| De la Haye et al. [15] | Cross-sectional (Australia)  Theoretical framework/model: not stated | n= 385 {network 1: 164, network 2: 108, network 3: 113}c (81% to 93%) | Year level 8 to 9 (12 to 15 yrs of age); 47.5% girls | Participant reported in school survey; a) Physical activity and screen time behavior of nominated close friends within year level (reciprocated and non-reciprocated), b) Number of received friendship nominations | Participant reported in school survey; a) Organized physical activity (hrs/wk & times/wk), b) Non-organized physical activity (hrs/wk & times/wk), c) TV/movie watching (hrs/day), d) Other screen time [internet, gaming] (hrs/day) | Demographics, weight status | a) Boys’ friend's organized physical activity associated with individual’s organized physical activity in 2 networks (+)[other network (n.s)]; Girls’ friend's organized physical activity associated with individual's organized physical activity in 2 network (+) [other network (n.s)], b) Boys’ and girls’ friend’s unorganized physical activity not associated with individual's unorganized physical activity (n.s), c) Boys’ and girls’ friend’s TV/movie watching not associated with individual’s TV/movie watching (n.s), d) Boys’ friend’s other screen time activities associated with individual’s other screen time activities in 1 network (+)[other networks (n.s)]; Girls’ friend’s other screen time activities associated with individual’s screen time activities in 3 networks (+), e) Boys’ friendship nominations associated with organized physical activity in 2 networks (+) [other network (n.s)]; Girls’ friendship nominations not associated with organized physical activity (n.s); Boys’ and girls’ friendship nominations not associated with unorganized physical activity (n.s); Boys’ and girls’ friendship nominations not associated with TV/movie watching (n.s); Boys’ friendship nominations not associated with other screen time activities; Girls’ friendship nominations associated with other screen time activities in 1 network (+) [other networks (n.s)]. |
|  |  |  |  |  |  |  |  |
| De la Haye et al. [43] | Longitudinal: 1 year follow-up (Australia)  Theoretical framework/model: Theory of Planned Behaviour, Self Perception Theory | n= 378 {group 1: 222, 92.9%; group 2:156, 90.2%} | Group 1: 13.60 ± 0.40 yrs of age; 47.30% girls , Group 2: 13.7 ± 0.40 yrs of age; 44.90% girls | Participant reported in school survey; MVPA of nominated best friends and friends hang out with most within grade (reciprocated and non-reciprocated) | Participant reported in school survey; MVPA (hrs/wk) | Demographics, socio-demographics | Friendship selection influenced by similarities in friend's MVPA and individual's MVPA (+); Friend's MVPA associated with individual's MVPA over time (+). |
|  |  |  |  |  |  |  |  |
| Denault & Poulin [39] | Longitudinal: 5 year follow-up (Canada)  Theoretical framework/model: not stated | n= 390 (75%) | 12.4 ± 0.42 yrs of age; 55% girls | Participant reported in school survey (Time 1); Sports participation of nominated three best friends within classroom (reciprocated) | Participant reported in school survey (Time 1-5) and researcher conducted phone interview (Time 3-5); Sports participation (hrs/wk, mths/yr) | Demographics, socio-demographics, parental variables, other | Boys’ friend's sports participation associated with individual's sports participation (+); Girls’ friend’s sports participation not associated with individual’s sports participation (n.s); Boys and girls friend’s sports participation associated with individual's sports participation over time (+). |
|  |  |  |  |  |  |  |  |
| Gesell et al. [40] | Longitudinal: 1.2 year follow-up (United States)  Theoretical framework/model: not stated | n= 81 (n.r.) | 7.96 ± 1.74 yrs of age; 65.40% girls | Researcher conducted face-to-face interview (Time 1,2,3); Physical activity of nominated friends within after-school program (reciprocated and non-reciprocated) | Accelerometer (Time 1-3); Physical activity (avg. daily count) | Demographics, weight status | Friendship selection and breakage not influenced by similarities in friend’s physical activity level and individual’s physical activity level (n.s); Friendship nomination and received friendship nomination not associated with physical activity level (n.s); Friend's physical activity level associated with change in individual’s physical activity level (+). |
|  |  |  |  |  |  |  |  |
| Jago et al. [31] | Cross-sectional (United Kingdom)  Theoretical framework/model: not stated | n= 986 (58.6%) | 10 to 11 yrs of age; 58.10% girls | Participant reported in school survey; Physical activity (counts per minute, MVPA) of nominated best friend of same gender within school (reciprocated and non-reciprocated) | Accelerometer; a) Physical activity (counts per minute), b) MVPA (counts per minute >2912) | Demographics, weight status, other | Boys’ and girls’ best friend's counts per minute not associated with individual's counts per minute (n.s); Boys’ best friend's MVPA associated with individual's MVPA (+); Girls’ best friend's MVPA not associated with individual's MVPA (n.s). |
|  |  |  |  |  |  |  |  |
| Livesey et al. [47] | Cross-sectional (Australia)  Theoretical framework/model: not stated | n= 192 (n.r.) | 10.75± 0.95 yrs of age; 58.3% girls | Participant reported in school survey: Rating of friend status (like to play) for all participants within class | Participant reported in school survey; Physical activity (mean activity score) | Demographics, other | Boys’ and girls’ friendship rating in play not correlated with individual's physical activity level (n.s); Boys’ and girls’ friendship rating not associated with individual's physical activity (n.s). |
|  |  |  |  |  |  |  |  |
| Macdonald-Wallis et al. [44] | Cross-sectional (United Kingdom)  Theoretical framework/model: not stated | n= 986 (58.6%) | 10 to 11 yrs of age; 55.8% girls | Participant reported in school survey: Physical activity (spatial correlation for counts per minute, MVPA) of nominated four closest friends within school (reciprocated and non-reciprocated) | Accelerometer; a) Physical activity (counts per minute), b) MVPA (counts per minute >2912) | Demographics, socio-demographics, other | Friendship network's counts per minute are similar to individual's counts per minute (+); Friendship network's MVPA are similar to individual's MVPA (+); Immediate through fifth degree friend’s MVPA and counts per minute associated with individual’s MVPA and counts per minute, respectively (+); Friendship network’s MVPA associated with individual’s MVPA (+); Friendship network’s counts per minute not associated with individual’s counts per minute (n.s). |
|  |  |  |  |  |  |  |  |
| Ommundsen et al. [45] | Longitudinal: 3 year follow-up (Norway)  Theoretical framework/model: not stated | n= 80 (67.8%) | 6 to 10 yrs of age (grades 1 to 4); 55% girls | Participant reported in school survey: Nomination of 3 friends prefer to work and play with most (Time 1 & 4) | Accelerometer; Physical activity (total counts) (Time 1) | Demographics, weight status, socio-demographic, other | Socio-metric status in grade 1 correlated with physical activity in grade 1 (-). Socio-metric status in grade 4 correlated with physical activity in grade 1 (+). For girls, socio-metric status in grade 4 associated with physical activity in grade 1 (+). For boys, socio-metric status in grade 4 associated with physical activity in grade 1 (-). |
|  |  |  |  |  |  |  |  |
| Raudsepp & Viira [41] | Cross-sectional (Estonia)  Theoretical framework/model: Social Learning Theory | n= 475 (81.30%) | 14.2 yrs of age (13 to 15 yrs); 50.95% girls | Participant reported in school and at home survey; Physical activity of nominated best friend | Participant reported in school survey; Physical activity (hrs/wk) | Demographics, socio-demographics | Boys’ best friend's moderate, hard, and very hard intensity physical activity associated with individual’s higher activity level (+); Girls’ best friend's MVPA not associated with individual’s higher levels of physical activity (n.s); Girls’ best friend's hard and very hard intensity physical activity associated with higher levels of physical activity for individual (+). Best friend's physical activity is a significant predictor of individual's physical activity (+). |
|  |  |  |  |  |  |  |  |
| Schofield et al. [42] | Cross-sectional (Australia)  Theoretical framework/model: not stated | n= 318 (92.10%) | 16.0 ± 0.80 yrs of age; 100% girls | Participant reported in school survey; Physical activity of nominated three closest friends within school in descending order (reciprocated and non-reciprocated) | Pedometer; a) Physical activity (< 10,000 or ≥ 10,000 steps/day) and step counts | None | Girls’ first through third nominated friend’s step count associated with individual's step count (+)*; Girls with one, two, or three active friends significantly more active compared to girls with no active friends (+); First and second nominated reciprocated friend's step count associated with individual's step count (+)*; Third nominated reciprocated friend's step count not associated with individual's step count (n.s)*; First and third nominated non-reciprocated friend's step count not associated with individual's step count (n.s)*. Second nominated non-reciprocated friend's step count associated with individual's step count (+)*; First nominated friend's physical activity associated with individual's physical activity (+)*. Second and third nominated friend's physical activity not associated with individual's physical activity (n.s)*. |
|  |  |  |  |  |  |  |  |
| Strauss & Pollack [46] | Cross-sectional (United States)  Theoretical framework/model: not stated | n= 17557 (79%) | Grade level 7 to 12 (12 to 18 yrs of age); 45 % girls | Researcher conducted in-home interview; Nomination of 5 best male and 5 best female friends within school | Participant reported in school survey; a) Sports participation (≤ 2 or > 2 times/wk), b) TV/video watching (hrs/wk) | Demographics, weight status, socio-demographics | Friendship nominations associated with sports participation (+); Friendship nominations associated with TV/video watching (-). |
|  |  |  |  |  |  |  |  |
| Yli-Piipari et al. [38] | Cross-sectional (Finland)  Theoretical framework/model: Expectancy-value model | n= 330 (80%) | 12.2 ± 0.22; 52.42% girls | Participant reported in school survey (Time 1); Physical activity of nominated three friends like to spend time within grade level (reciprocated and non-reciprocated) | Participant reported in school survey (Time 2); Physical activity (days/wk) | Demographics | For boys, within peer group showed homogeneity for physical activity (+)*. For girls, within peer group showed homogeneity for physical activity (+). |

| *Note.* Associations between social network and physical activity/sedentary variables are considered statistically significant at p < 0.05 unless otherwise stated (i.e., *= p < 0.10 or “n.s” = not significant). |
| --- |
| (+) = a positive association between social network and physical activity/sedentary variables.  (-) = a negative association social network and physical activity/sedentary variables. |
| MVPA= moderate-to vigorous-intensity physical activity.  n.r. = not reported.  Confounder categories: *Demographic* [age, gender, race]; *Weight Status* [height, weight, body mass index, weight status]; *Socio-demographics* [parent SES/income, education level, pocket money]; *Parental variables* [parent community involvement, parent beliefs about organized physical activity]; *Other* [pubertal status, behaviors, self-efficacy, movement assessment and motor skills, autonomy]. |
|  |
